# What are the challenges when recruiting to a trial in children’s social care? A qualitative evaluation of a trial of foster carer training

**DOI:** 10.1186/s13063-021-05186-9

**Published:** 2021-04-01

**Authors:** Gwenllian Moody, Lucy Brookes-Howell, Rebecca Cannings-John, Sue Channon, Elinor Coulman, Alyson Rees, Jeremy Segrott, Michael Robling

**Affiliations:** 1 Centre for Trials Research, Cardiff University, Neuadd Meirionydd, Heath Park, Cardiff, UK; 2 Children’s Social Care Research and Development Centre (CASCADE), School of Social Studies, Cardiff University, Cardiff, UK; 3 Centre for the Development and Evaluation of Complex Public Health Interventions for Public Health Improvement (DECIPHer), Cardiff University, Cardiff, UK

**Keywords:** Looked after children, Foster Care, Social care, Fostering Changes, Confidence in Care, Out-of-home care, Trial recruitment

## Abstract

**Background:**

Randomised controlled trials (RCTs) are comparatively rare in UK social work, but can offer distinct advantages. Confidence in Care (CiC) is an RCT with embedded process evaluation evaluating Fostering Changes (FC), a 12-week training programme for foster and kinship carers to increase skills and coping strategies. In order to mitigate challenges in participant recruitment, an engagement strategy was designed to maximise this. Our aim is to explore experiences of key study stakeholders towards trial recruitment and identify broader messages about recruitment to social care trials.

**Methods:**

Three focus groups were conducted, two with field-based recruiting staff (*n* = 7) and one with carers who attended the FC programme (*n* = 8). Five interviews were conducted with trainers who delivered FC, eight with foster carers who attended the programme, 18 with Foster Carers who elected not to take part in the programme, and 12 with social workers from participating trial sites. In addition, an away day for FC trainers was observed and discussions related to recruitment were noted. Transcribed audio-recorded data were inductively coded, double-coded by a second researcher, and thematically analysed.

**Results:**

Six themes were identified. The first addressed pragmatic aspects of the intervention affecting recruitment (e.g. committing to a 12-week programme). A second focussed on accuracy of communication about the trial between provider agencies and carers. A third concerned the ability of recruiting staff to contact carers, a particular challenge in group-based recruitment. A fourth addressed trial methods and their communication (e.g. relationship between trial team and recruiting staff). A fifth explored lack of differentiation by carers between the roles of the various professionals (e.g. FC facilitators and provider agencies). The sixth addressed perceived differences between recruitment into social care and health studies.

**Conclusions:**

Recruitment challenges in this social care setting were similar to those in healthcare. Some (e.g. gatekeeping by professional staff) may be rooted in randomisation anxiety, or unfamiliarity with research methods. Researchers more familiar with healthcare recruitment were however encouraged about the experience of working in this care setting. The original recruitment strategy and adaptations form the basis of further recommendations for research practice.

**Trial registration:**

ISRCTN19090228. Registered on 11 January 2017.

**Supplementary Information:**

The online version contains supplementary material available at 10.1186/s13063-021-05186-9.

## Background

Randomised control trials (RCTs), widely considered to be the gold standard for assessing effectiveness [1–3] and common in healthcare evaluation, are less frequently used to evaluate social care interventions in the UK. Reasons suggested for their relative scarcity in social care include being viewed by some social care academics and practitioners as unethical, particularly in relation to the concept of randomisation [1, 3–5]. Recruitment to RCTs can suffer if practitioners or organisations who are involved in supporting recruitment view RCTs with suspicion [3].

### Recruitment challenges in RCTs

Recent RCTs in children’s social care in the UK have faced recruitment challenges and so struggled to recruit sufficient participants to draw strong conclusions about intervention effectiveness (e.g. [2, 3, 6]). Recruitment is a ubiquitous challenge in trials not solely in social care [7]. Difficulty in recruiting to trials can increase overall cost and bias samples, leading to smaller samples and underpowered studies [8], resulting in a lack of high-quality evidence for social care policy.

### The Confidence in Care RCT

The Confidence in Care (CiC) Trial evaluated Fostering Changes (FC), a 12-week group-based training programme for foster and kinship carers to increase skills and coping strategies [9, 10] (ISRCTN19090228). The programme was commissioned to run with a group size of 12 carers, a mix of foster and kin carers. Week one of the training programme is an induction session and thereafter each session lasts 3 h and consists of last session feedback and skills covered the previous week, a review of the theoretical material underlying the topics for that week, new skills/strategies to be used at home, and end of session feedback. Participants were randomised to either receive FC or usual care. The FC programme was delivered by four delivery partners independent of the research team: The Fostering Network, Barnardo’s, Action for Children, and *The Adolescent and Children’s Trust,* each with a team of trainers. The FC programme ran in local authorities (LAs) and independent fostering providers (IFPs) in Wales between January 2016 and April 2017 with some sites delivering more than one training course in this period. The FC programme was delivered both within and outside the CiC trial. Recruitment occurred in waves aligned to the school terms (three rounds of recruitment are planned per year). Each LA supplied the trial team with a list of pseudonymised foster carer details who met study eligibility criteria. For each site/wave, at least 50 carers were randomly selected to be invited to take part. Both LAs and IFPs further provided to the trial team a subset of at least 18 eligible foster carers considered to be both interested and suitable to take part in the trial, who had provided consent to be contacted by the trial team. Foster carers were contacted by Health and Care Research Wales (HCRW) Researchers or a member of the core trial team for recruitment and the completion of baseline measures at a home visit or during a telephone interview.

#### Study aim

We undertook a qualitative process evaluation to explore experiences of key study stakeholders towards trial recruitment and potential broader messages about recruitment to social care trials.

## Methods

### Trial participant recruitment strategy

An external pilot phase run at two sites, in which there was poor early recruitment, informed changes to the trial recruitment strategy which are listed in Table 1. This table outlines the original method, the change that was made and the timing of this, and the reason for the change.

**Table 1:** Changes made to trial recruitment strategies

Change to recruitment strategy	Timing of change	Original method	New method	Reason for/consequence of change
*De-coupling baseline assessment from allocation*	Before recruitment began, including at pilot sites.	Participants assessed at baseline and allocated to FC or usual care group immediately afterwards.	Baseline assessment was conducted first and at a later date participants were remotely allocated to group.	The change allowed a greater run-in time within which to conduct recruitment. Formerly all recruitment had to occur over a short period of time immediately prior to groups starting.
*Adjusting the allocation ratio from 1:1 to 2:1*	After the pilot phase and before the second wave of recruitment.	Participating carers allocated in equal numbers across the two study groups, maximising statistical power.	Allocation ratio changed to 2:1 to FC and usual care and usual care only group. This design had less power, meaning that the overall sample size was increased.	A minimum number was required in the trial arm to reach the required group size of 12 foster carers. Changing the allocation ratio increased the likelihood of filling an intervention group where total numbers of participants at a site are restricted (i.e. a group of twelve could be formed with only 18 recruited participants in this approach, whereas, 24 would be required using a conventional 1:1 allocation).
*Increase the period of time during which recruitment could be undertaken prior to groups running*	After the pilot phase and before the second wave of recruitment.	During the pilot service providers and foster carers were approached approximately 6 weeks before the programme was due to start.	Approach time was increased to up to 4 months.	This allowed more time for service providers and Trial team to contact foster carers and for foster carers to respond. It also gave foster carers more notice of the possibility of attending the 12-week programme.
*Improved participant materials*	The materials used in the initial approach were revised between the pilot phase and before the second wave of recruitment.	The original documents were designed to fit the established processes for clinical trials	The first approach letter was amended after piloting to contain less text and to be more reader-friendly in the formatting. A reminder leaflet was introduced designed to be posted out to foster carers 1 week after the initial approach letter to remind them of the Fostering Changes Programme and the Confidence in Care evaluation.	The aim was to produce a simpler, briefer and more accessible document set. This may reduce a barrier to engaging initially with the study. The original documents were designed to fit the established processes for clinical trials where often participation risks can be higher (e.g. new drug treatments) and coercion possible so they are designed to protect patients. However in the present trial, we considered that some streamlining was possible without compromising individual’s ability to form an informed decision to participate.
*Service providers’ selection of foster carers as well as all foster carers invited (change from all foster carers invited only)*	After the pilot phase and before the second wave of recruitment.	For each site/wave, at least 50 foster carers were randomly selected by the trial team to receive a study pack from the LA. Foster carers registered their interest by responding to this.	In addition to the original process, service providers (LAs and Independent Fostering providers (IFP)) provided a subset of at least 18 eligible foster carers considered to be both interested in and eligible for the trial, and who had provided permission to be contacted by the trial team. Provider agencies selected participants to nominate based on locally determined criteria, including perceived needs of a foster carer, or apparent availability based on absence of competing commitments.	To better target eligible foster carers who might be interested.

To recruit participants in the main trial phase, each LA supplied the trial team with pseudonymised details for foster carers meeting study eligibility criteria. At least 50 carers were randomly selected to receive a study invite containing study information and details of how to register an interest to attend. Both LAs and IFPs also provided a subset of at least 18 eligible carers considered to be both interested and eligible to take part in the trial, who had provided permission to be contacted by the trial team. Recruitment was completed by either HCRW researchers or a member of the core trial team.

During the trial, HCRW researchers were trained in recruitment and data collection on two occasions. The first session delivered by FC trainers introduced the HCRW staff to the role of foster caring; the second session addressed further changes to recruitment strategies and was run by the trial manager.

### Qualitative study on recruitment

Recruitment into the trial was explored through focus groups, interviews, and an observation exercise with key stakeholders as part of a broader process evaluation. This publication focuses on recruitment and the data was collected for a broader process evaluation of the trial [11].

### HCRW researchers

Two focus groups were conducted in June to August 2017 with HCRW researchers. Each HCRW staff member who recruited into the trial was invited to take part via email. The aim of the focus groups was to explore the experiences of professional researchers recruiting participants to the trial, specifically how participants were recruited, successes and challenges, perceived differences in approach compared to other, mostly healthcare studies that they recruited to, support for their role by the trial team, and their reflections on recruiting to social care trials. The first group contained three participants, and the second contained four participants.

During the CiC trial, some additional data collection was undertaken to gather the reasons for non-recruitment of foster carers. HCRW researchers maintained a log following every contact attempt to potential participants.

### Foster carers

A focus group was conducted in April 2016 with eight foster carers who attended the FC programme in one LA in South Wales. These carers were recruited as part of the trial; however, due to low numbers, this group was reassigned as a pilot group and participants withdrawn from the trial. The aims of the focus group included exploring their thoughts on the provision of services and support for foster carers in Wales and their opinions of the FC programme, including of any facilitators or barriers to taking part in both the intervention and the trial.

All foster carers who had attended a FC programme were contacted via email and letter and invited to take part in individual interviews to explore their experiences of, involvement in, and attitudes towards the FC programme. Eighteen telephone interviews were conducted between September 2017 and April 2018. Eight telephone interviews with foster carers who elected not to take part in FC were undertaken between September 2017 and April 2018, to explore foster carer engagement with formal training programmes, including their attitudes towards the FC programme and recruitment into the trial. These were recruited from a list of approached but non-participating foster carers who had agreed to be contacted further.

### FC trainers

Five interviews were conducted in November to December 2017 with FC trainers. The trainers all delivered the FC programme within the trial (and also to non-trial groups). An email was sent to each of the delivery partners inviting their trainers to take part, and at least one trainer from each of the four partners participated*. The aim of these interviews was t*o explore enrolment into the FC programme both within and outside the trial. *Four interviews were conducted face-to-face and one was conducted on the telephone.* An away day was undertaken by the delivery partners for FC trainers which focussed on various aspects of their work including any challenges relating to both enrolment to the FC programme and recruitment to the trial.

### Social workers

Twelve interviews with social workers were undertaken between September 2017 and April 2018. An email was sent to staff responsible for coordinating recruitment at LA fostering teams and IFPs inviting social workers involved with trial recruitment and enrolment to the FC programme to take part. The aim of these interviews was to explore the experiences of, involvement in, and attitudes of social workers towards the FC programme. Eleven interviews were conducted via telephone, and one face-to-face. The majority of participants were qualified social workers. Four participants were social workers at management level and one participant was not a qualified social worker, but worked in the training team that organises training for foster carers.

### Ethics

The trial was approved by Cardiff University School of Social Sciences Research Ethics Committee (ref. no. SREC1515). All participants provided written informed consent and were made aware they could withdraw their participation at any time.

Semi-structured interview schedules and topic guides were developed by the research team informed by the research aims and existing literature (Additional file 1). All focus groups and interviews were conducted by members of the trial team with previous qualitative experience (GM, SC, LB-H, JS), or by master’s level student social workers supervised by members of the research team.

### Analysis

Each of the groups and interviews were digitally recorded and anonymised transcripts were created.

An inductive methodology, thematic analysis, was used to analyse the data and followed the methods recommended by Braun and Clarke [12]. A coding framework was devised by a member of the trial team and validated by another. Validation was completed by reviewing at least 15% of the data to determine if the coding framework was a suitable description of the data and transcripts were double-coded until consensus was reached. This involved two researchers independently coding the same pieces of data and checking that they had both applied the codes in the same way. Data analysis was supported by using NVivo (version 11).

## Results

Table 2 contains details of the interviews and focus groups.

**Table 2:** Details of the interviews and focus groups

Key stakeholders	Details
**HCRW Researchers** focus group (total HCRW researchers attached to the trial: 15)	2 groups (3 and 4 participants) Group 1 132 min, group 2 92 min
**Foster carers** (Total recruited to the trial: 312, total declined: 137)	
Focus group (trial pilot group)	8 participants 76 min
Interviews with foster carers who took part in FC	60 contacted, 18 agreed to interview. 14/18 female, 16 LA 2 IFP 3/18 kin carers Years of experience range 1.5 - 26 (median 7) Between 30 and 60 min each
Interviews with foster carers who elected not to take part in FC	161 contacted 8 agreed to interview 7/8 female 7 LA 1 IFP 8/8 non-kin carer Years of experience 2.5 - 27 (median 13) Approx. 40 min each
**FC trainers**	
FC trainers interviews	4/5 female Range 28 – 55 min
FC trainers away day	15 trainers, facilitated by 2 Fostering Network staff and 1 FC intervention developer 88 min
**Social workers** interviews (17 LAs and 2 IFPs were included in the trial)	8/12 female 7 LA 5 IFP 7 social workers 4 social work managers 1 training team manager Approx. 60 min each

Drawing on all data sources, six principal themes were identified and are described below.

### Intervention content and delivery

Both the HCRW researcher group and the foster carer group discussed how practical challenges of attending the FC programme may be a barrier to recruitment. Challenges included time commitment, travel, childcare, and venue-related issues. Some of these challenges may have been particularly pertinent for foster carers as a population as they were felt to be time-poor.

HCRW researcher (focus group) (Participant 5, group 1): And they’d got a lot of other meetings and the children had particular issues. How quickly their diary fills up with their various different meetings …

Foster carer who chose not to be part of trial (interview) (Participant 1): It was always going to be on a Wednesday. That was no good because I work every Wednesday.

Some foster carers believed that the intervention was more suitable for newer carers.

Local social workers joined some groups as participants, a change to the original FC model. The attendance of social workers on the FC programme was felt by both HCRW researchers and foster carers to impede recruitment. Some were concerned about being able to be completely open about their experiences of fostering for fear that any disclosures would not be kept confidential. In practice, although some admitted to being initially apprehensive about the presence of a social worker, this had actually been a good experience.

Foster carer who attended the programme outside the trial (focus group) (Participant 1): Myself, I had reservations about (social worker) being here and being able to speak freely because there’s been massive issues around confidentiality, and might have, you know, you have that thing in the back of your mind well, if you’re being honest. Is it being taken back to the office?

Some of the HCRW researchers felt that the offer of other training programmes affected foster carers’ decision to take part in the trial and the FC programme. Concerns were voiced by some carers that the FC programme would not complement other courses. Foster carers felt that they could not commit time to attending both the FC programme and other training courses. Many foster carers however had told the HCRW researcher that they were keen to take part in the FC programme as they felt that it offered something that other courses did not.

HCRW researcher (focus group) (Participant 1, group 1): Because some of them had some courses and they felt that they didn’t really deal with the issues that they had to deal with. And I suppose, depending on your child you’re going to have different issues cropping up at different times. So yeah it was quite … people were quite willing to take the [yeah] the course really.

Some HCRW researchers voiced concerns about FC programmes that were running outside the trial and the ethical implications of this for foster carers being randomised to the control arm.

Social workers mentioned that programme eligibility criteria may have been barrier to engagement and recruitment, particularly those regarding placement length and the requirement to have a child on placement during the course which can be difficult to predict.

Social worker (interview) (Participant 10): So that's been the tricky bit really in that sometimes they got a placement when they start and then they haven't. …So some people felt miffed that they, you know came to the initial session and they couldn’t continue because they didn't have a placement. So those issues were kind of difficult to resolve, erm initially, erm and so that was difficult as well in terms of buy in when people weren't able to participate because of that.

### Service providers

There was widespread concern from HCRW researchers and FC trainers that information provided by service providers to both themselves (through the trial team) and to foster carers was either incorrect or insufficient. This theme was discussed extensively. HCRW researchers were also concerned that some foster carers were told that attendance of the programme, and recruitment to the trial, was mandatory. Information about the course and the trial was received by foster carers via different sources (for example, their social worker, a link worker) and different routes (for example, an email, a phone call) and that the information varied in its emphasis (for example, as an opportunity, as mandatory, or as research).

HCRW researcher (focus group) (Participant 5, group 1): The numbers that we had, they weren’t all eligible either [right] and you’d have people [right] that didn’t have children [yeah], details, so the wrong contact details, the wrong … they’d moved. It was just … so, the details that we were sent from, and meant to be from the social workers, the contact details were wrong as well.

HCRW researcher (focus group) (Participant 1, group 2): There was one that definitely said that she’d been basically told to go on the course.

There were some concerns voiced by FC trainers that enrolment to the FC programme was not a priority for service providers.

FC trainer (Away day) (Participant 4): For example, we had a big issue……coming up with the right numbers. I spoke to the fostering team manager and in the end I got really annoyed and I just said look, if you can’t get me 12 people by next week I’ll be writing to your head of service. And that afternoon I had 12 people. Just got really annoyed…….it was just they weren’t prioritising.

Establishing local contacts and links between social work teams and both recruiters and FC trainers was seen as very important and useful to both FC trainers and recruiters, and HCRW researchers as this was seen as a key to better recruitment. It was felt that more collaboration between the aforementioned stakeholders at the very beginning of the trial could have circumvented some of the above issues.

FC trainer (interview) (Participant 1): I think the biggest thing and from what we’ve seen in terms of success………..actually being involved upfront, before they’ve named their families, in order that they are meeting us, they get to ask us direct questions, they’re meeting other people who’ve done the course, and so they have a greater insight into what they’re actually coming into, so then that’s probably the biggest improvement.

Many of the social workers described the process and decision-making as a management decision as to who was put forward and that this was disconnected from other members of the team.

### Reaching foster carers and group recruitment

HCRW researchers felt that contacting foster carers to arrange interviews was a challenge, in particular if the carer had not been pre-informed by their service provider that they would be contacted. Some researchers adapted recruitment processes slightly to meet this challenge.

Interviewer: What kind of things did you learn do you think?

HCRW researcher (focus group) (Participant 6, group 1): I think it’s the way of contacting people, you know, no replies and then us starting to send out Participant information Sheets just so we make sure that even if we call them a second [time] that they didn’t think there was harm in that. Cos, often you put something down when you’re busy. You need to read it and then you completely forget about it, which happens in every household including my own.

Informed consent and data collection could be completed over the telephone. As this was a new way of working for most of the HCRW researchers, there were some concerns regarding the ethics of this method (although this had been approved). After using this method however, most were very happy with it, including the practicalities and time saved from not having to travel across large geographical areas to recruit participants at home. Some even felt that foster carers preferred this method as it was less intrusive than home visits.

HCRW researcher (focus group) (Participant 3, group 1): No, I think probably, now, I was resistant myself to the telephone contact at first. Afterwards, now I’d say that that was a better system.

Some HCRW researchers experienced difficulties with *group* recruitment, that is, recruiting sufficient foster carers to get requisite numbers of participants in a FC group, mainly on account of unfamiliarity with this. Both the HCRW researchers and FC trainers felt pressure to fill groups.

### Trial methods and their communication

HCRW researchers reported challenges with communication between themselves and the trial team, particularly with regards to amendments made to the trial’s recruitment strategy

HCRW researcher (focus group) (Participant 5, group 1): (One of my colleagues) was saying study design changes were a little concerning earlier on. It seemed that the field was dictating the study method and approach which she felt was very uncomfortable. They just changed, if we didn’t get recruits in the first phase, then, alright, we’ll lower the number of recruits. They changed the questionnaire we did. The questionnaire went smaller all of a sudden. And that was between phases. And she was really concerned about that.

There was a feeling amongst FC trainers that foster carers recruited to the *trial* had better knowledge of the FC programme than those who attended the FC programme outside the trial, and that they were also more likely to be eligible to attend the programme as eligibility had already been ascertained in the trial setting.

There were reports from both HCRW researchers and FC trainers that both foster carers and service providers expressed confusion around and disliked the concept of randomisation. This was one of the most common themes discussed in these groups. For example, many foster carers wanted to attend the FC programme with people they knew and were unhappy when uncertainty was expressed about if this would be possible. In spite of some issues around randomisation, HCRW researchers reported a general feeling of positivity from foster carers about the trial. The randomisation process was not well explained to foster carers by social workers and this made some reluctant to take part in the trial. Some foster carers described being “chosen” and one foster carer, who had this understanding, equated it to being special and then felt this was a misrepresentation of the actual process. This was more to do with communication than being unhappy at being randomised.

Foster carer who attended the programme (Interview) (Participant 3): (My agency ) put it over like you’d been chosen, so it made you feel a bit special and then when you find out down the line that wasn’t actually the fact, you weren’t chosen as it were, it was just the fact that everybody was going to get the opportunity to go, it was half the people would be chosen for this year that’s just … gone and then the other half of the people would do it in the next year, one that come up, so there was a little bit of deceit there on their part.

The randomisation process was also problematic for some social workers. This was in part influenced by their subsequent role in the group or recruitment. Some had been involved in engaging foster carers in the training before the trial commenced so the introduction of a different approach was noted. The process of randomisation caused some disappointment in the team when foster carers considered to be in need were perceived to have “missed out”. Keeping foster carers who were allocated to the control group interested in attending 1 year on was also seen as a problem. Some of the social workers had really not been happy to—as they saw it—relinquish their usual control over who attended and who did not.

Social worker (Interview) (Participant 11): Erm, and then it was like from there we were told then who was going to be attending and who was going to be on the control group so. I did say in one of the meetings I was a bit disappointed because especially one of the carers, err we felt she would really benefit and she ended up chosen on the control group. …And there was a bit of discussion around well, you know it's not supposed to be crisis training, and I was trying to say I'm not saying it's crisis, it's just that when you know a placement is struggling any support you can put in is good.

Social worker (Interview) (Participant 9): I think that frustrated us, to be honest, and them, because they were desperate to get on it. We thought we had the place for them, and um, and then didn't.

### Who is who?

Another emergent theme was the lack of understanding by foster carers of the various professionals’ roles from the FC programme (trainers), the CiC trial (recruiters / researchers), and service providers (social workers).

HCRW Researcher (Focus group) (Participant 6, group 1): And you arrived, they would open up more about their problems and their issues which was something that I found quite hard because we were not that trained for social workers and the problems that they were facing. It was, it was quite difficult I found.

### A social care RCT

HCRW researchers reported feeling very positive about the experience of working on a social care study, rather than the much more common health care studies in their workloads. It was felt like there had been more focus on health studies in general and not enough was done to support social care studies.

HCRW researcher (Focus group) (Participant 4, group 1): … a bit keener I think as a team, or some of us in the team are keen to [yes] support more social care research. Our managers certainly are actively encouraging us to …

HCRW researcher (Focus group) (Participant 3, group 1): We could do with more.

HCRW researcher (Focus group) (Participant 4, group 1): … to support social care. We just need to have the studies chucked at us.

Some of the ethical aspects of the trial however were sometimes viewed as quite lax, as compared to health studies.

HCRW researcher (Focus group) (Participant 2, group 1): And the ethics, I suppose probably. Because of the change suddenly it seems like anything was allowed.

HCRW researchers felt there were fewer gatekeepers in this setting and so gaining access to potential participants was different from a healthcare trial.

### Reasons given for non-recruitment

During the CiC trial, some additional data collection was undertaken to gather the reasons for non-recruitment of foster carers. HCRW researchers maintained a log of reasons for non-recruitment of foster carers (Table 3). The most common reason for not wanting to take part was being unable or unwilling to attend a 12-week course, which may elude to time commitment issues.

**Table 3:** Reasons recorded by recruiters for trial non-recruitment

Reason	Number of carers ***n***/%
No reason provided	43 (20.7%)
Uncontactable	25 (12.0%)
Course full/started/cancelled	12 (5.8%)
Ineligible	30 (14.4%)
Does not feel they would benefit from course/not interested in course	9 (4.3%)
Trial-related reason	1 (0.5%) (did not feel incentive was good enough)
Cannot attend the 12-week course	88 (42.3%)
TOTAL	208

## Discussion

To explore how recruitment to a social care trial was experienced by key stakeholders, we completed interviews, focus groups, and an observation exercise to enable multiple perspectives to be represented. Six themes were identified. The first addressed aspects of the intervention affecting recruitment (e.g. committing to a 12-week training programme). A second focussed on accuracy of communication between provider agencies and carers. A third concerned the ability of recruiting staff to contact carers, a particular challenge in group-based recruitment. A fourth addressed trial methods and their communication (e.g. the relationship between trial team and recruiting staff). A fifth explored the lack of differentiation by carers between the roles of the various professionals (e.g. FC facilitators and provider agencies). The sixth addressed observations by stakeholders of differences between recruitment into social care and healthcare studies.

### Recruitment challenges

Recruitment challenges faced by both Dixon et al. [2] and Mezey et al. [3] were also found in the CiC trial, including anxieties about randomisation from various stakeholders. Randomisation can be viewed as the unfair withholding of an intervention that is seen as beneficial (even if no evidence may exist for this) [2, 3]. Some changes were made by Dixon et al. [2] and Mezey et al. [3] to address poor recruitment during their trials. Some similar changes were made in the CiC trial, for example increasing the period of time during which recruitment could be undertaken prior to groups running. The full list of changes in the CiC trial is outlined in Table 1.

Trial recruitment is a methodological priority in healthcare and other settings (e.g. the PRioRiTy (Prioritising Recruitment in Randomised Trials) study [13], the MRC Trials Methods Research Partnership group on Trials Conduct [14]).

There were common issues in the Dixon et al. [2] and Mezey et al. [3] trials and the CiC trial surrounding support from service providers and managers, some of which may have also partly been rooted in randomisation anxiety. Although some randomisation anxiety was observed, it is worth noting that access to the FC programme was available initially to carers via the trial only (due to limited training capacity in Wales), but that control arm participants were offered access to it at end of trial period.

Another very strong common theme was a concern that service providers did not provide foster carers with enough and/or the correct information about the FC programme and the trial. Two priorities in the PRioRiTy study concerned communication with the public: the information that should be communicated to improve recruitment and the best approaches for designing and delivering this information. The randomisation anxiety experienced by foster carers and service providers in the CiC trial may have been allayed to some extent if improvement and clarifications were made to the way information about randomisation was conveyed. Some other concerns noted in the current study about the information passed from service providers to participants also echo these priorities.

Features of the intervention and its delivery were a barrier to recruitment in the CiC trial, as well as the aforementioned RCTs, for example practical challenges to attendance. This reflects another priority in the PRioRiTy study which was to question the motivators that influence members of the public’s decisions to take part in a RCT. The most common reason given for non-recruitment of foster carers into the CiC Trial was time commitment issues. It should be noted however that there were some feelings that the FC programme offered something that other courses did not. The FC programme was selected by the consortium of delivery partners from a shortlist of programmes following stakeholder research [15]. It was not reported that either stakeholder group (young people or professionals) in this research discussed duration of the programme and the logistics of attendance. As Oakley et al. [4] suggest, the personal significance attributed to an issue by participants is particularly important in social interventions.

We found a lack of understanding by potential participants of the various professionals’ roles. The extent to which carers were unable to clearly distinguish the roles of service providers, intervention providers, and recruiting research staff may have implications for how they appraise and respond to research requests. Such confusion is ethically harmful and may be practically damaging too if messages about the trial (for example, about voluntariness of participation) are being interpreted partly on the basis of the perceived role of the informant. If future studies anticipate this to be a potential problem, they may be able to address the issue with study materials that clarify professionals’ roles.

Reaching targets for group recruitment was challenging, and HCRW researchers reported responding with a flexible approach within the boundaries of ethical approval. Communication between the trial team and HCRW researchers was a challenge, particularly regarding amendments made to the recruitment strategy and the perceived ethics of telephone recruitment. Researchers more familiar with National Health Service [NHS] (ethical review) were initially wary of operating within a different governance model (e.g. University ethical review for a non-clinical study). In practice, they found the approach acceptable and facilitative and reported feeling very positive about the experience of working on a social care study. The use of telephone recruitment reflected two of the priorities in the PRioRiTy study which were concerned with approaches to optimise informed consent, and the advantages and disadvantages of use of technology during informed consent. Similar adjustments in approach may be required to support future social care studies. Clear communication at the outset between trial teams and recruiters informed by our experiences may reduce tension and allay concerns while still running such trials to high-quality standards.

Better routes for engaging lay input are needed in this particular kind of trial in children’s social care. We did utilise a pilot group as an initial lay contact group and subsequently brought in a lay rep to Trial Steering Group to provide higher level PPI. It may be the case that obtaining earlier and more consistent PPI input would have been helpful; however, we found this difficult to get in place early on.

Despite these challenges, recruitment to the CiC Trial was successfully achieved with the target sample size surpassed [10], although changes were made to the trial to address poor early recruitment (Table 1), including adjusting the allocation ratio from 1:1 to 2:1.

### What went well

Aspects of recruitment into the trial that went well included the introduction of consent and data collection via telephone, and HCRW researchers reported feeling very happy to utilise this method after initial reservations. As mentioned above, the experience of working on a social care study was a positive one for HCRW researchers and there was a clear sense that more such studies should be conducted. Another successful aspect of recruitment was that FC trainers felt that those recruited to the trial had more knowledge of the FC programme as compared to their non-trial counterparts, which may potentially impact intervention adherence. With regard to the process evaluation of the trial, there was diverse representation from a relevant range of stakeholders, and a variety of methods (interviews, focus groups, observation and record logs) were used to have multiple but complementary perspectives on same issues. Many foster carers took part in the qualitative data collection but this did not involve those allocated to the usual care arm. The data for this publication was collected for a broader process evaluation of the trial which addressed both arms of the trial. This was a RCT and there should be no difference between recruited carers allocated to the two study arms (as it was at random), and therefore, feedback on recruitment (e.g. deciding whether to take part in the study) should not suffer by only having those in intervention arm. However, it should be noted that those in the intervention arm were also more likely to be followed up in the trial; this may mean that that that group were on the whole more engaged, and therefore maybe also more likely to be positive about other aspects of the study (including thoughts about recruitment). By including those who were approached but chose not to participate in the study, we attempted to sample those who were less enthusiastic about the study as well as those who were more enthusiastic. Utilising social work students to conduct interviews meant early exposure to working on a trial for these students who will be future practitioners. Utilising students may also have been associated with good engagement from social workers and foster carers.

### Lessons learnt

Establishing local contacts, i.e. the early and intensive involvement of specific individuals from each service provider, acting as a champion of the programme, was seen as an important facilitator to recruitment and an improvement that could have been made to the trial. Although the trial team established relationships by meeting with teams from each service provider (including a named individual who would be the contact regarding recruitment at that site) early on in the recruitment process, HCRW staff were not involved in these meetings. In the CiC trial, the trial team acted as the ‘go between’ between service providers and HCRW researchers, and although more could have been done to improve communications between all parties, it is still felt that it was imperative that the trial team were kept ‘in the loop’ with regard to recruitment progress at each site. Similar future work should focus on supporting face-to-face meetings between HCRW researchers, FC trainers, and service providers to foster a better understanding of each other’s roles and goals. Improvements should also have been made to communications between the trial team and HCRW researchers on amendments to the trial and in particular to recruitment. Although communication via email was sent out to all HCRW researchers involved in the team responsible for recruitment into the trial when changes were made to trial processes, this communication was perhaps too one-way and attempts should have been made to meet with the researchers to discuss any concerns they had regarding these. It may also be the case that the trial team underestimated the difference in experience of HCRW researchers between health and social care trials. This may be in part because the trial team’s experience lay mostly with complex interventions compared to the medical or drug trials normally worked on by HCRW researchers.

In our trial, heads of service were engaged with and supportive of the trial but greater attention to teams on the ground may have been productive. Embedding a researcher to recruit participants in social work teams could be explored, although given the number of teams involved in this study it would have been very resource intensive. The use of social workers to recruit participants in other RCTs presented challenges (e.g. [2, 3]), for example, Dixon et al. [2] found that some social workers preferred to rely on professional judgement when selecting children to take part in the study rather than all who were eligible. The utilisation of professional researchers, such as HCRW, for recruitment is strongly recommended in future such trials, especially as they gain greater familiarity of the care environment and become more familiar to social workers. Even so, service providers may still act as ‘gatekeepers’, i.e. the trial team were reliant on social workers for inviting eligible foster carers and ensuring that they provided their consent to be contacted by the trial team, and thus any trial would need their full commitment going forward.

During the trial, the majority of recruited carers had been identified directly to the research team as both interested and in agreement to be approached as opposed to solely being sampled from the long-list of carers provided by sites. A number of factors may account for this. This may, for example, reflect a more personalised initial approach from service teams to carers than possible by contact from the research team. Similarly, service teams may also have a greater understanding of actual eligibility, availability and likely interest and which is not initially known to the research team. In practice more than one route to approaching potentially eligible trial participants may be possible and blending them may be desirable. What will be key is ensuring that any proposed model supports equity of access, does not reduce generalisability (for example, by unduly narrowing the pool of carers included), and is research efficient (for example, minimising resource and staff burden while maximising likelihood of recruitment). It is also essential that the voluntary nature of trial participation is clarified and maintained across all recruitment paths and at all stages of communication ensure public rights.

### Social care RCTs

The running of a social care social care study may be somehow ‘different’ to running a health care study, partly due to differences in governance procedures, for example, in making amendments. The levels of professional training and experience in research of professionals are also likely to be key factors in the easy at which these sorts of trials can be conducted. It should be kept in mind however that similar barriers to recruitment are found in both social care and health care studies [7].

There was a clear sense that more social care RCTs should be conducted, particularly from the HCRW researchers. Other researchers (e.g. [3]) have complained of a resourcing issue for social care research, and historically, infrastructure funding has been more plentiful for healthcare trials. Increasing the number and quality of studies in social care, including centres driving the use of RCTs, is imperative to ensuring a better evidence base and recommendations for practice.

## Conclusions

Recruitment challenges in this study were similar to those in other social care RCTs and indeed in many healthcare studies. Some of these anxieties may be rooted in randomisation anxiety, or unfamiliarity with research methods. The original recruitment strategy and adaptations form the basis of further recommendations for research practice.

## Supplementary Information

**Additional file 1.**

## Data Availability

The datasets used and/or analysed during the current study are available from the corresponding author on reasonable request but which would require additional processing to ensure confidentiality.

## Abbreviations

RCT: Randomised control trial
CiC: Confidence in Care
FC: Fostering Changes
LA: Local authority
IFP: Independent Fostering Provider
HCRW: Health and Care Research Wales
PRioRiTy Study: Prioritising Recruitment in Randomised Trials Study
NHS: National Health Service
