# Supplementary material for: What are the challenges when recruiting to a trial in children’s social care? A qualitative evaluation of a trial of foster carer training
Source: Trials. 2021 Apr 1;22:241. doi: 10.1186/s13063-021-05186-9 (PMC8015028; doi:10.1186/s13063-021-05186-9)
Supplement: Supplementary file 1 — Additional file 1. [file 13063_2021_5186_MOESM1_ESM.docx]

**Appendix 1: Focus Groups and Interviews Topic Guides**

**HCRW Researchers** focus group

**Overall aim:** To explore experiences of professional researchers recruiting participants to the Confidence in Care Trial

- Broadly what is your current role / background (eg, professional background, types of study, functions in role – maybe not solely recruitment for example, training for your role)

**CiC specific**

- What has been your in role on the Confidence in Care study?
  - [High level] any management responsibility, general description
- What would recruiting participants to the study have involved for you?
  - E.g. narrative description of process of recruitment in CiC from their perspective (including practical aspects / considerations such as time incurred / travel, forms etc)
  - As it may arise prompts about face-to-face / telephone recruitment
- Your successes and challenges in recruitment
  - What did you find facilitated recruitment into this study – however big or small that was?
  - What did you find to be more challenging about recruiting to this study – again however big or small?
  - How / did you modify your approach to recruitment for this study / during this study?
  - Specific probes:
    - Were there specific concerns mentioned / identified by approached carers that may have put them off taking part?
    - Were there aspects of the study or the intervention that was appealing to the foster carers?
- Possible differences in approach
  - How did recruiting on the phone and in person compare – and what were the pro & cons? [Assume that this is applicable – i.e. that some individuals did both, or at least that between individuals in the FG that there was experience of both?]
  - Were you aware which carers had been selected by LAs and which had not [ie those shortlisted / contacted by SWs and those responding to the general mailshot] – and if so how did that affect your approach / experience?
  - How did recruitment into this study (any aspect of the process and experience) compare to recruiting into other studies that you have worked on?
    - Were there any aspects of this study that were particularly different, challenging, easier?
- Completing the baseline CRF (if this hasn’t emerged from previous sections)
  - How did participants respond to the questionnaire – did it seem acceptable?
  - Were there any difficulties with it? Particular questions or comments from participants?
  - Was there anything you noticed about the question asking for the three main behaviours they wanted to tackle? How did participants approach it? How did you deal with this?
  - How long did it usually take to complete?
- How have you been supported in your recruiting role in CiC?
  - how has the initial study documentation, briefing and training helped?
  - How did the follow-on training (with input from the social work / facilitators) help with your role?
  - Would any other preparation / support / training have been helpful?
  - Could any changes to the materials or processes used by you in CiC have either helped you or participants?

**Social Care studies on the portfolio**

- What are your thoughts about HCRW recruiting to social care studies?
  - Have you been working on other social care studies?
  - Do you think you will have other social care studies in the future?
  - Is there anything you have learned from working on CiC that would be useful to help your team or others recruit to social care studies? [Maybe will have been covered in relation to CiC preparation / support / training ideas]

**Foster carers**

Focus group (trial pilot group)

**Fostering Changes programme**

1. What has been your experience of the Fostering Changes programme?

- Do you see it as something that foster carers need?

1. Was the programme telling you anything new, or was it reinforcing what you already know?
2. Was it useful?
3. How would you improve it?
4. Having been through the Fostering Changes programme can you think of anything it offered that might appeal to other foster carers?
5. When you were asked if you wanted to take part in the programme, was there anything you were told that made you definitely want to do it?
6. What might put other foster carers off doing the programme?
7. Recruitment: SC give overview of current recruitment process. How can we encourage other foster carers to take part in the Fostering Changes programme?

**Your needs as a foster carer**

1. What are the things you **need** to support you in your role as a foster carer?
2. Do you **get** that support that you need in your role as a foster carer?
3. What types of support and training do you actually get as foster carers? e.g. Social worker, other formal support / training, informal support (e.g. friends/family), anything else?
4. What do you *not* get that you need?
5. Are there other types of support available that you don’t use, perhaps that other carers use?
6. What is the most/less important support/training you have had?
7. How do you find out about different types of support/training available?
8. How would you improve the support/training you receive?
9. Taxonomy of support/training

Interviews with foster carers who took part in FC

Expectations:

- What were you hoping Fostering Changes would give you? Did it do this?

Overall Reflections:

- What were the most useful things you got from FC?
- What if any are the main differences now in your approach to being a foster carer compared to a year or so ago? Has this been influenced by Fostering Changes?
- What would you say are the main messages of Fostering Changes for foster carers? (maybe the things you think you might remember in a year or twos time)

Implementation

- Were there particular activities or sessions you remember that were the most helpful?
- How did you find the experience of working in a group of foster carers?
- What were your thoughts about the ‘homework’?
- Did a Social Worker attend your FC group? If yes…
  - Were they your social worker?
  - How did having a social worker in the group with you make a difference?
- Were there things you remember as being unhelpful? Any tricky times? [Probe: Have you used any of the strategies your learnt in practice – how did that go?]
- Have you been to any of the follow-on support groups? What was that like?
- Did you feel the course had content suitable to support foster carers with children of all ages? Did you notice anything particularly relevant for teenagers?
- Did the group run as you expected it to?
- Did the facilitators seem to be following a plan? Did they share the plan with you?
- Were you confident in the facilitators?

Changes in behaviour and thoughts:

- Did it change the way you do things with your foster children [probes: ‘… at home’ ‘… in relation to school’
- Did it have any effect on how you feel about yourself as a foster carer? [key probe: For example to your confidence – if so in what way?]
- Did it change how you think about your foster children’s behaviour?
- Did it change how you think about your behaviour with them? [Probe: did it make you feel like you were more likely to succeed in doing what you set out to do with them? ]
- Have the children you look after noticed a difference do you think? Have they said anything that makes you think “oh yes..that was what we learned on fostering changes”

Other courses:

- Have you done any other courses to support your role as a foster carer?
- How does Fostering Changes compare? How is it similar? How is it different?
- If you met a new foster carer what would you say to them about the different courses? What would you recommend they do (and in what order)
- Is there anything you think should be offered that isn’t? Any courses you have heard about that you haven’t been offered?

Processes of recruitment and participation

- Who contacted you and what information did they provide?
- Did you have as much information as you needed to make a decision about taking part?
- What did you think about taking part in the trial?
- What did your social worker say about taking part in Fostering Changes?
- Did your social worker say anything about taking part in the trial?
- Can you remember the questionnaire you had to fill out? Was there anything you particularly remember about filling it in? Was it reasonably straight forward ? Any tricky questions? Anything you had to miss out or wanted to pass over?

Other support

- What types of things do you find support you as a foster carer?
- Do you get any particular types of support e.g. social worker, information, group support etc
- Any services that you use which offer that support with particular difficulties e.g. health, mental health, education

Interviews with foster carers who elected not to take part in FC

Experience

- How long have you been a foster carer?
- How many children/ young people have you fostered in that time?
- Do you have any children/ young people with you at the moment?
- Are you registered with an independent fostering agency, or the local authority?
- Did you have an induction, or period of training to prepare you for the role when you

first became a foster carer?

- Do you see foster caring as a profession? *(explore view))*

Fostering Changes Training

- Who initially contacted you to take part in the Fostering Changes training?
- How did you feel about being contacted in this way/ by this person?
- What do you remember about the information that was provided to you when you

were approached to take part in the Fostering Changes training?

- Did you have as much information as you needed to inform your decision about taking part?
- How did you feel about taking part in the training?
- What were your main reasons for not taking part in the training?
- Although you did not attend, have you heard anything about the Fostering Changes

training from other people?

Foster Carer Training

These questions aim to explore opinions on foster carer training in general.

- How are you normally informed about training opportunities?
- How do you feel about current training opportunities for foster carers in your area?
- Which courses have you completed to support your role as a foster carer?
- What would influence you to want to take part in a training programme?
- What makes a training programme good? *e.g. location, course leader, new*

*information etc.*

- What makes a training programme bad?
- Are there any courses that you have been offered but turned down? *(if yes, then could you please explain a little bit about your decision?)*
- Do you feel that there are any topic areas or subjects that aren’t covered within the training that is available for foster carers?
- When you attend a training programme, what are you hoping to gain from the

experience?

- Is the professionalisation of foster care linked to training?
- How do you feel about taking part in training which is also attended by social workers?

Support

These questions aim to gain opinions on the support that is available to help foster carers attend training programmes

- What support do you receive as a foster carer? *(e.g. childcare, breaks, peer support,*

*travel costs etc.)*

- Does this support have any impact on your ability to attend training programmes?
- Do you receive regular supervision with your social worker?
- Is training a subject that is approached during supervision?
- Does your social worker have an impact on your decision to attend certain training

programmes?

- Is there any support that you feel you should receive, but is not currently available?
- What could be put in place to help support foster carers to attend training

programmes?

**FC trainers**

FC trainers interviews

**Overall aim:** To explore experiences of Fostering Changes Facilitators in running the Fostering Changes programme.

- Broadly what is your current role / background (e.g. professional background, accreditation)
- As well as Fostering changes what other courses for foster carers have you delivered? (recently or in the past)
- (For TACT staff only) Did you deliver Fostering Changes before the Confidence in care roll out? (so before January 2016?)

**About the programme**

- How to do feel the Fostering Changes programme fits in with other training programmes you might have heard of or delivered that are being run by Local Authorities for Foster Carers?

(Prompt: do you feel the Fostering Changes programme fills any gaps that other programmes may not provide, or do you feel that it conflicts in terms of ethos or advice compared to other programmes?)

- Has delivering fostering changes meant that other training programmes have been dropped from the timetable of training?
- How well prepared have you felt to deliver the Fostering Changes programme?
- Have you gone for accreditation to deliver the programme? Why did you choose to do so? Do you feel that being accredited to deliver the programme has enabled/will enable you to be better prepared?
- What do you consider to be the main goals of Fostering Changes ?

(Prompt - what do you expect foster carers to get out of it? What changes would you expect

to see as a result of attending the programme?)

**Before the group starts**

- Do you have any knowledge of the communication about the programme before the programme starts between Local Authority or IFP and Foster Carers? Do you think the most appropriate information is being passed to Foster carers? Would you suggest any changes or improvements in how this is done?
- What communication do you have with the Local Authority about the group before it starts?
- In some Fostering Changes programmes the facilitator meets with each Foster Carer in their home before the programme starts. In Wales this is replaced by the group induction session. Did you have an induction session? Was this separate to session 1? What do you think are the main purposes of that induction session? What do you think about the idea of the initial one-to-one meeting?

**Running the group**

- There was some discussion about time management during running the group at the facilitator day in July 2017. What are your thoughts on time management in the group?

(Prompt: ways of keeping on topic, managing individual versus group needs, when people haven’t done the home practice?)

- When running the programme are there any aspects that were difficult to deliver or you felt like you needed to adapt?
- Did you have Social Workers in your group? What do you think were the advantages and disadvantages of this (if they attend)?
- What do you think about the various compositions of the group – there can sometimes be a mix of Foster Carers with children of various ages, as well as Foster Carers with varying experience. How do you think these things affect the group?
- How about the differences in foster carers who offer, or don’t offer, respite care?
- Are there any differences between the needs in the group of standard Foster Carers and kin or family carers?
- Did you deliver the 12+ module? How did that go?
- Were there aspects of the programme that went especially well? Tell me about the strengths of the programme.
- Has delivery of the programme changed as you have got more experienced? (if yes – in what ways?)
- Are there any aspects of the programme you would like to change if you could?

(prompt materials, structure , numbers of carers and numbers of sessions etc.)

- Tell me what you think about the support groups?
- Has running Fostering Changes affected the way you deliver other training with foster carers?

(Prompt – have you used any of the ideas or materials in your other training?)

**Trial specific**

- Did you run both trial and non-trial groups?

If yes:

- - - Did you find the process of recruitment easier or harder to non-trial groups?
    - When running the groups, were there any aspects of the trial groups that were particularly different and if so in what way?

**Social workers** interviews

- Could you tell me a little about your role?
- How long have you been in this role?
- How many foster carers that you work with have done the Fostering changes course?
- Before we start talking about Fostering Changes I would like to get a clearer picture about the types of support foster carers get from different sources. What support and what courses would they usually access? (prompt – anything else…..)
- Could you take me back to when you first heard about the fostering changes course and the CIC trial and tell me what did you know about the training, at this time?
- What were your expectations for the training? Use of prompts- once participant has spoken – What did you feel were the aims of the training? What did you think foster carers would gain from it ? Was the training of interest to you?
- What did you know about the CIC trial, at this time? (prompt - what did you think about the idea of running a trial?)
- Did you play any role in recruiting foster carers to the trial? Could you tell me about your experience of doing this?- use of prompts once participant has spoken- What went well? Were there any challenges? How did the team manage it? What was the general response by foster carers?
- What motivations do you feel that foster carers have in attending the training? –Use of prompt once participant has spoken- Did you have to persuade any of them? Did foster carers receive any incentives to attend the training? - Dependent on the local authority, foster carers are required to ensure they attend a certain number of training hours each year to remain registered as foster carers. It would be interesting to find out if there is any pressure by the recruiting social worker on foster carers to maintain professional standards by attending training. It would also be interesting to know if social workers selected participants based on their training needs as identified by foster carers or social workers during their supervision. What did you think the foster carers were hoping to get out of it?
- Did you attend any of the training sessions yourself? (How many and which ones?)
- Could you tell me how you came to attend? (prompt if needed re team process - was it their idea, volunteer or requested by manager, selected etc)
- How did you find the training? – use of prompts once participant has spoken- What do you remember about the training? Were there any significant learning points for you? Was there anything you didn’t like about the training? Was there anything that could have been better?
- Can you tell me how you felt about how the training was delivered? (Use of prompts – good venue? Materials? Facilitators skilled in running groups? )
- Did any of the foster carers that you work with also attend the training with you?
- Can you tell me how you felt about this? What was it like being on the same course?
- What was it like being a Social Worker on the training with foster carers generally?
- How do you feel the training was received by the foster carers you attended the training with? – Prompt once participant has spoken- How did the group get on together? How did they get on with the training? What was the impact of you being there for Foster Carers?
- What do you think about the training? Prompts once participant has spoken- Has the training contributed anything to your professional practice? Do you feel the training has contributed anything to the foster carers that have received it? Did you think it met the needs of foster carers of children with different ages?
- What would you say are the main outcomes that the Fostering Changes programme is aiming to achieve?
- (Depending on answer to previous question) In the previous study of Fostering changes and in the current trial one of the main outcome being measured is carer efficacy. Do you think this was something the training might help with? (If asked what is meant by this, say “for example confidence that they know what to do to help their foster child, making a difference to their future, that type of thing)
- Now that the programme has been running for a while I wondered if you thought it had made any difference to any other parts of the service? (Prompt maybe some of the ideas or exercises have been included in other training or you have dropped other training because fostering changes is running for example)
- Do you envision any role for this training moving forward? Prompts once participant has spoken- Have you come across any training like the fostering changes programme? Do you have any thoughts on how the training could be organised in the future? – i.e. who leads the organisation of the training? when the training takes place and for how long? (i.e. timing of the training and number of sessions), where the training could take place? (i.e. location), how the training influences group dynamics? Would you make any changes to the training? –> topics covered? delivery methods?
- What other training do you feel that foster carers are in need of? Are there things you think they need that they don’t currently get?
